# Design and Validation of a PLC-Controlled Morbidostat for Investigating Bacterial Drug Resistance

**DOI:** 10.3390/bioengineering11080815

**Published:** 2024-08-10

**Authors:** Adrián Pedreira, José A. Vázquez, Andrey Romanenko, Míriam R. García

**Affiliations:** 1 Biosystems and Bioprocess Engineering Group (Bio2Eng), Spanish National Research Council (IIM-CSIC), Rúa Eduardo Cabello 6, 36208 Vigo, Spain; apedreira@iim.csic.es; 2 Group of Recycling and Valorization of Waste Materials (REVAL), Spanish National Research Council (IIM-CSIC), Rúa Eduardo Cabello 6, 36208 Vigo, Spain; jvazquez@iim.csic.es; 3 CIENGIS Lda, Rua Pedro Nunes, 3030-199 Coimbra, Portugal; andrey.romanenko@ciengis.com

**Keywords:** morbidostat, programmable logic controller (PLC), adaptive laboratory evolution (ALE), didecyldimethylammonium chloride, *Escherichia coli*, bioreactor

## Abstract

During adaptive laboratory evolution experiments, any unexpected interruption in data monitoring or control could lead to the loss of valuable experimental data and compromise the integrity of the entire experiment. Most homemade mini-bioreactors are built employing microcontrollers such as Arduino. Although affordable, these platforms lack the robustness of the programmable logic controller (PLC), which enhances the safety and robustness of the control process. Here, we describe the design and validation of a PLC-controlled morbidostat, an innovative automated continuous-culture mini-bioreactor specifically created to study the evolutionary pathways to drug resistance in microorganisms. This morbidostat includes several improvements, both at the hardware and software level, for better online monitoring and a more robust operation. The device was validated employing *Escherichia coli*, exploring its adaptive evolution in the presence of didecyldimethylammonium chloride (DDAC), a quaternary ammonium compound widely used for its antimicrobial properties. *E. coli* was subjected to increasing concentrations of DDAC over 3 days. Our results demonstrated a significant increase in DDAC susceptibility, with evolved populations exhibiting substantial changes in their growth after exposure.

## 1. Introduction

Understanding the mechanisms driving antibiotic resistance is crucial to address one of the major human health concerns, according to the World Health Organization: the rise of multidrug-resistant pathogens [1]. In this context, morbidostats represent a sophisticated and innovative class of continuous culture systems designed to perform adaptive laboratory evolution (ALE) experiments in the presence of antibiotics and other antimicrobial compounds. Through ALE, researchers can observe and analyze, online and in vitro, microbial adaptation and evolution under environmental stressors, including the presence of antimicrobial agents.

A morbidostat operates by dynamically adjusting the concentration of an antimicrobial agent to maintain a predefined growth rate or optical density of the microbial population. This is achieved through a feedback loop wherein the system continuously monitors the culture’s growth parameters and modulates the antimicrobial concentration accordingly. A typical morbidostat setup consists of multiple mini-bioreactors, each equipped with optical sensors for online monitoring of culture density. A computer-controlled feedback system, interfaced with peristaltic pumps, adjusts the flow rates of antimicrobial solutions and fresh media. The algorithm employed by the morbidostat ensures that the microbial population is perpetually exposed to sub-inhibitory but challenging antimicrobial concentrations, promoting the selection of resistant phenotypes through time.

The architecture to control morbidostats has improved since the original design by Toprak et al. [2], although it still presents some limitations. Some designs use a custom MATLAB application running on a Windows desktop machine for data recording and pump control [2,3,4]. However, as noted by Nguyen et al. [5], this architecture presents certain limitations. MATLAB’s timer class is unsuitable for real-time applications running on standard operating systems. As stated in the MATLAB documentation, “the timer object is subject to the limitations of your hardware, operating system, and software. Avoid using timer objects for real-time applications” [6]. Other morbidostat designs involve using Arduino for data acquisition and a customized Python application running on a desktop machine for process control [7,8]. Although inexpensive and fully operational, Arduino has limited processing power and memory, lacking the robustness and reliability of industrial-grade solutions such as a programmable logic controller (PLC). Furthermore, both MATLAB and Python languages may experience garbage-collector events, which can lead to stop-the-world pauses with unpredictable duration in the application, unacceptable for time-critical tasks, such as flow controllers.

Here, we present a PLC-controlled morbidostat design with a focus on data validity and real-time control of the process. Data acquisition and pump control are centralized in a PLC, ensuring quick and predictable execution times. Both the database and the Supervisory Control and Data Acquisition (SCADA) software run in a real-time operating system (Linux machine with low-latency real-time kernel). The device is further validated by evolving *Escherichia coli* by employing didecyldimethylammonium chloride (DDAC), a widely used disinfectant, as a stressor. Such a hybrid setup combines the robustness of a PLC with the flexibility and extensibility of general PC SCADA systems, together with the possibility of running more complex supervisory algorithms from higher-level languages, such as MATLAB or GNU Octave.

## 2. Materials and Methods

**Figure A1:**
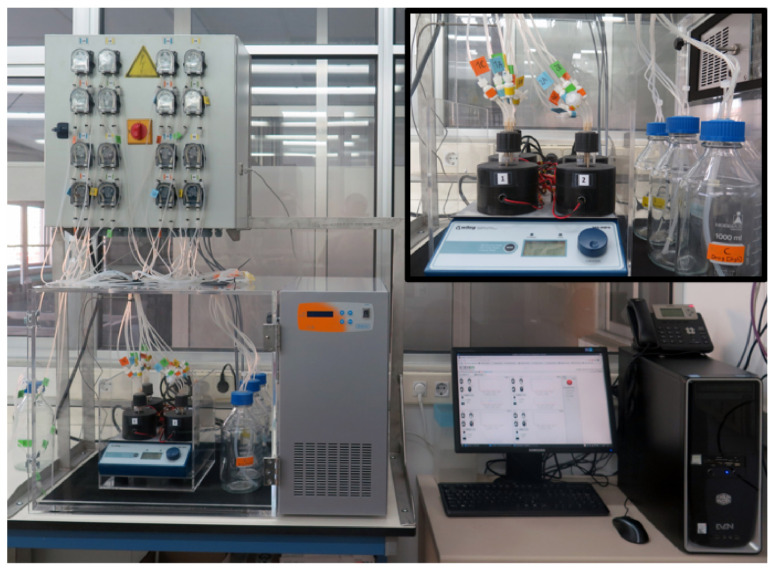
Photography illustrating the morbidostat setup, including the fludic system, the incubator, the control box, and the associated desktop computer.

The morbidostat setup presented here (Figure A1) has been designed to ensure precise control and monitoring of microbial cultures when studying adaptive evolution to antimicrobials. The following subsections include a detailed description of the morbidostat design, as well as the experimental procedures to run the system.

### 2.1. Morbidostat Design and Building

#### 2.1.1. Control System

**Figure 1:**
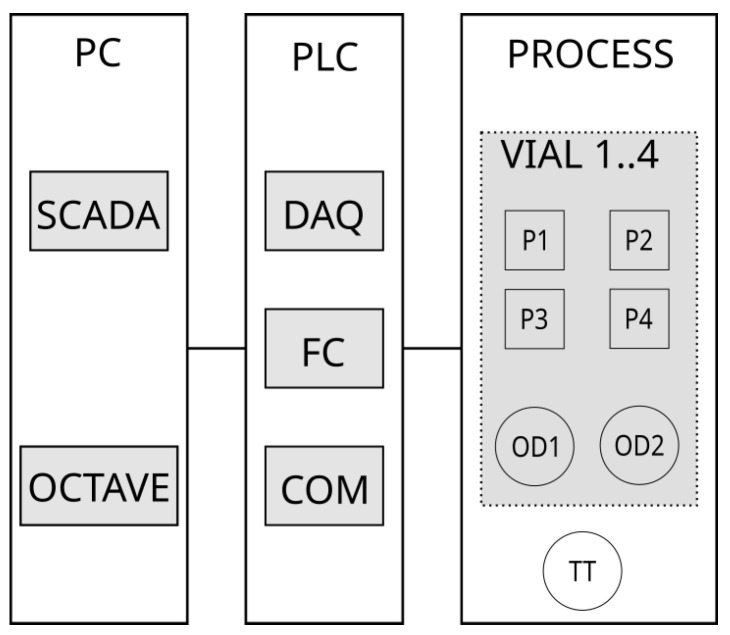
System overview.

The overall system features a processing unit, an electronic control enclosure, and a personal computer, as depicted in Figure 1, containing electronic components necessary to carry out the measurement, control, actuation, and networking of the system. More details are provided below.

##### Processing Unit

From a measurement and control point of view, the processing unit consists of four bioreactors (for clarity, only one is depicted in the ‘PROCESS’ block in Figure 1), each of them equipped with two phototransistors (SFH 300 FA-3/4, OSRAM GmbH, Munich, Germany) that measure scattered infrared light in the vial produced by an LED emitter (LD 271, OSRAM GmbH, Munich, Germany). Each reaction vial has four peristaltic pumps operated at 24 VDC. Additionally, the temperature in the vicinity of the four vials is measured with a PT100 probe. More information about the experimental setup is provided in Section 2.1.2.

##### Programmable Logic Controller (PLC)

The PLC subsystem performs three main operations: data acquisition (DAQ) (including signal conditioning to match the impedance of the phototransistors with that of the analog input module of the PLC), flow control (FC), manipulating the ON–OFF pumps via pulse width modulation (PWM) according to one of the algorithms described below, and finally, communication [COM] that provides two independent Modbus TCP server connections. Typically, one connection facilitates data exchange with the SCADA system, and the other may be used by another program such as MATLAB or GNU Octave.

The execution of the periodic measurement and control activities is performed in a VIPA SLIO 015 PLC that allows industrial-grade real-time control of the experiments, which otherwise cannot be assured using typical desktop hardware and software, even when special measures to lower the latency are in place [9]. The SLIO PLC is equipped with 0-10 VDC analog input modules that receive the measurement signal from the infrared photodetectors that measure the optical density (OD), capturing the scattered infrared light generated by the emitters. In order to match the impedance of the photodetectors with that of the analog input modules, an interface circuitry based on operational amplifiers is placed in between. Since each vial has two photodetectors, it is possible to configure the system at the PLC program level to use either of the photodetectors or their average value for measurement and control. In order to filter measurement noise caused by stirring and other factors, a low pass filter is implemented in the PLC program. The corresponding filter parameters, together with the photodetector calibration constants, may be readily adjusted from the user interface for each of the vials.

The PLC system is also equipped with a set of digital output modules that perform flow-rate control by modulating the pulse width of 24 VDC peristaltic pumps via a block of interface electro-mechanical relays, according to the following:

(1) $$F=F_{\mathrm{nom}}\frac{t}{T},\;s.t.\;t\leq T,$$

where *F* is the resulting flow rate, $F_{\mathrm{nom}}$ is the nominal flow rate of the pump, *t* is the time the pump is ON, and *T* is the period. The choice of *T* is a balance between the smoothness of the flow rate and the ON–OFF relay wear in order to achieve it.

Although the 12 pumps are the same, each of them can be calibrated individually based on the installed characteristics of each fluid line, and the corresponding constants are available in the graphical user interface.

**Figure 2:**
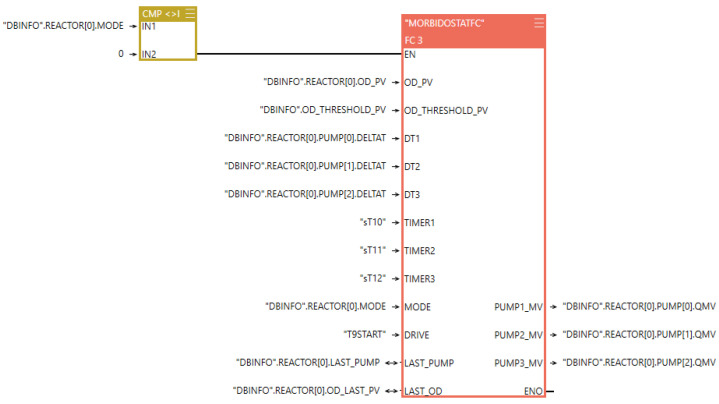
PLC program example segment.

The above-described functionalities are implemented in FBD and STL languages using the Speed7 Studio programming environment. As an example, Figure 2 illustrates a program segment that performs Pump 2 and 3 control.

**Off.** All control actions are disabled in the PLC. The system remains in an open-loop or may be controlled via the second Modbus interface, for instance, via MATLAB or Octave.

**Chemostat.** The controller operates as an open-loop system, assuring a predefined flow rate of the media that results in a certain residence time irrespective of the microorganism concentration inside the vial. The dilution rate (*D*) in the chemostat mode is as follows:

(2) $$D=\frac{F}{V},$$

where *F* is the flow rate (volume entering, same as volume leaving after mixing time, per unit of time), and *V* is the volume of the culture vessel. A steady state is achieved when D is equivalent to the specific growth rate (*µ*) [10,11]. This can be easily obtained since the dynamics of the population numbers are as follows:

(3) $$\frac{dN}{dt}=\left(\mu -D\right)N,$$

which is the derivative zero when D=μ.

**Turbidostat.** The controller operates as a closed-loop system regulating the culture based on OD measurements, which serves as an indirect indicator of cell density. As shown in Equation (4), when the OD surpasses a predefined threshold ($OD_{Thr}$), the system adds a predefined volume of fresh medium (Δ*V*) to the vial. After some mixing time, the same volume of old media is removed through the waste line, keeping *V* constant.

(4) $$OD>OD_{Thr}\to V=V+\Delta V.$$

**Figure 3:**
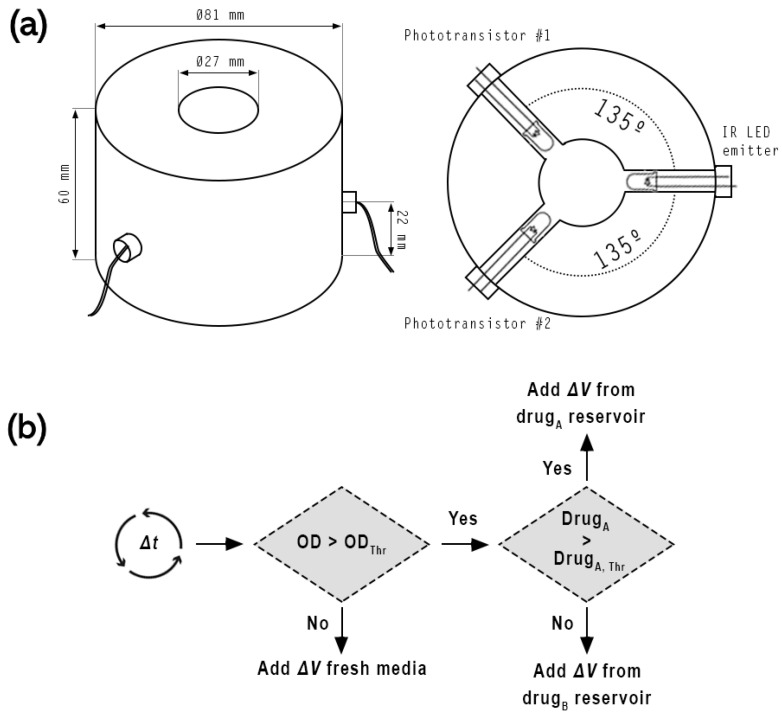
(**a**) Schematic representation of the vial holder and optical subsystem. The body of the holder consists of a mechanized polyoxymethylene cylinder. The infrared LED emitter and the phototransistors are positioned at an angle of $135^{\circ }$ to each other to maximize the detection of scattered light. (**b**) Control algorithm of morbidostat mode.

**Morbidostat.** The controller operates as a closed-loop system employing OD as a measured variable in a manner analogous to that of the turbidostat. However, the bacterial density is maintained below the threshold by adjusting the drug concentration in the culture vial (instead of diluting the media). Figure 3 depicts the morbidostats control algorithm. OD is evaluated every predefined period of time (Δ*T*). If OD is below the threshold, the culture is supplied with a Δ*V* of fresh growth medium. Otherwise, the system adds a Δ*V* of a drug-containing growth medium. Initially, this addition will come from drug reservoir “A” (containing growth media + the drug at a low concentration). However, if, after a certain number of attempts, the additions from this reservoir fail to reduce the OD of the culture, the system will be switched to drug reservoir “B” (with a higher drug concentration).

Note that the implemented algorithm in Morbidostat mode adds a drug whenever OD is greater than the threshold. However, in the original work by Toprak [2], the drug was added when both (1) OD exceeded the threshold and (2) the bacterial density was growing (positive OD slope). As discussed in the literature [11], Toprak’s algorithm cannot control the system when OD has strong inertia and a few additions of the drug are not able to trigger the decrease in the population. In that case, bacterial density begins to decrease at ODs much larger than the threshold, adding drugs only when the population diverges even more from the threshold, but never to return to the threshold.

##### Personal Computer (PC)

**Figure 4:**
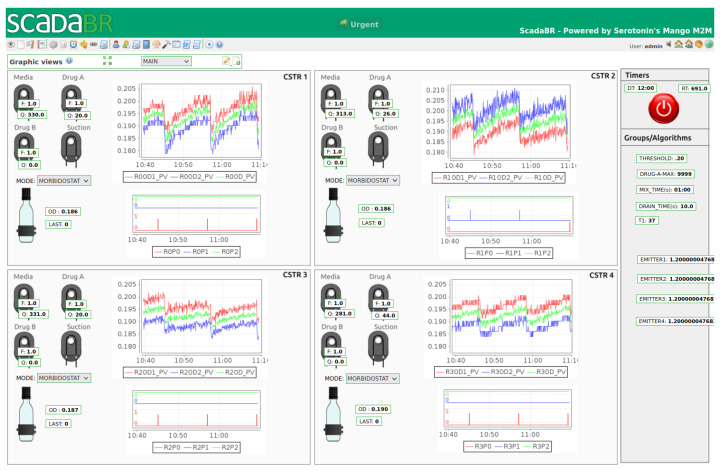
Graphical user interface provided by SCADABR.

The off-the-shelf PC runs a Linux Mint Vera 21.1 operating system, patched with a modified low-latency kernel, and hosts Supervisory Control and Data Acquisition (SCADA) based on the SCADABR open-source package (https://www.scadabr.com.br/), accessed on 4 December 2021. Its function is to provide a graphical user interface (GUI), as depicted in Figure 4, in order to visualize the data and process parameters, to manipulate thresholds, calibration constants, and the state of the pumps in manual mode, and to store and export data for post-experiment analysis.

It is noteworthy that the web-based GUI is accessible with a generic web browser from the PC itself or from other local or remote computers using secure authentication and authorization. The SCADABR instance runs as a Tomcat application and communicates with the PLC via Modbus TCP industrial protocol.

In addition to running the SCADA system, the PC also provides GNU Octave or MATLAB to run optional programs to perform more complex data acquisition or control, for instance, to manipulate the thresholds or setpoints of the flow controllers in the PLC, taking into account the multivariable and non-linear nature of the process as well as its long-time constants and pure delay. Similarly to SCADABR, the Octave program uses Modbus TCP protocol, and the two systems may be used simultaneously.

The resulting control system setup is of hybrid nature and allows for bringing together the real-time/low-latency performance and robustness of the PLC and the flexibility and openness of PC-based software systems, resulting in the following advantages:

- It satisfies real-time, low-latency performance requirements via PLC and ensures that the system will continue operating with the latest available setpoints, even if SCADA or MATLAB become unavailable (crash, network problems, etc.);
- It provides a cost-effective and feature-rich SCADA for data visualization, supervisory control, and data historicization and features web access from other computers if needed;
- Additionally, it allows us to use Octave/MATLAB for more complex data acquisition or control approaches (for instance, model predictive control [11], adaptive control law [12], and/or online state and parameter estimation);
- Using MODBUS TCP standard industrial protocol, it allows for eventual integration in other, more complex setups.

#### 2.1.2. Fluidic System

**Figure A2:**
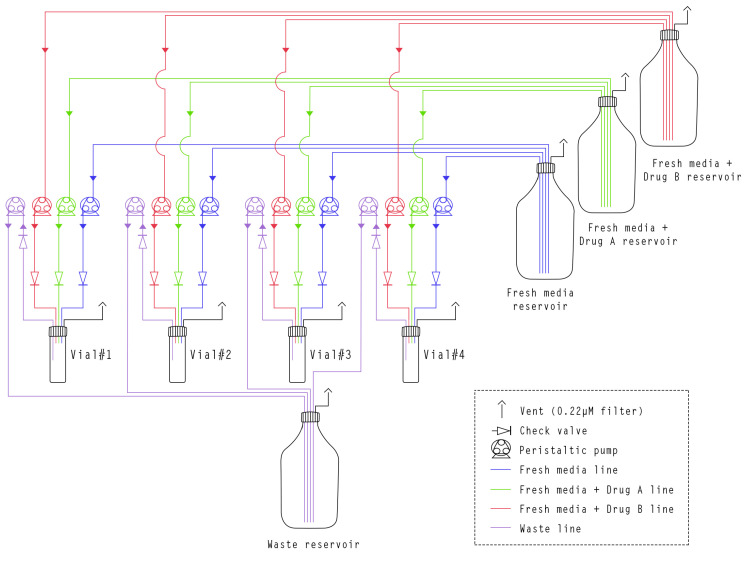
P&D diagram showing the fluidic system of the morbidostat.

The fluidic system comprises all the necessary components for the growth of microbial cultures and the transfer of different culture media. This includes culture vials, reservoirs, peristaltic pumps, and the connections between various components. Figure A2 provides a detailed schema of the interconnections between various components of the fluidic system.

**Figure 5:**
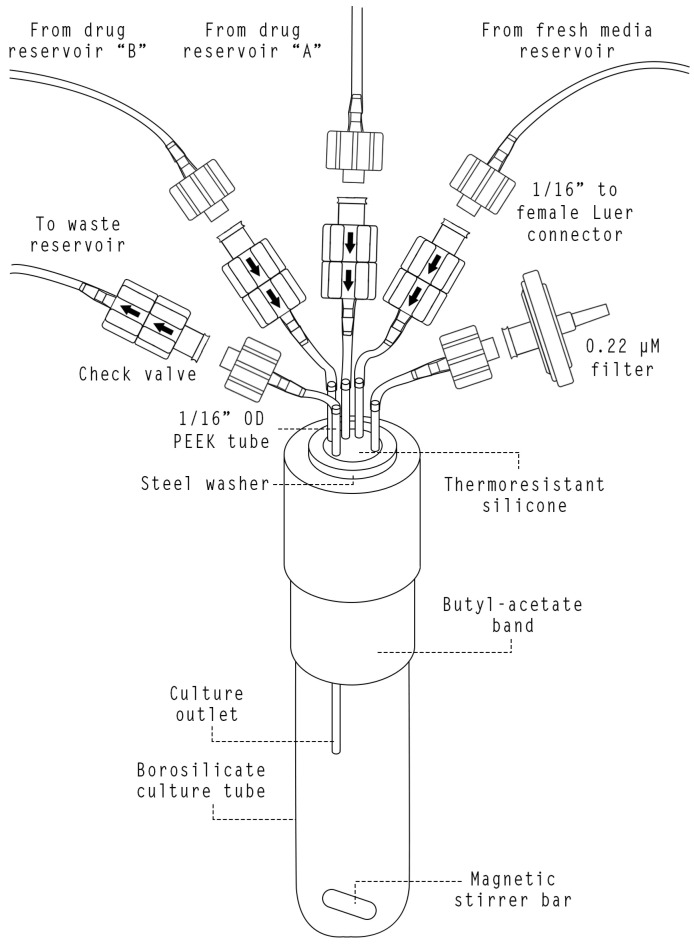
Schematic illustration depicting the different components of morbidostat vials.

Growth chambers (Figure 5) were built employing borosilicate culture vials (Pyrex™ 1636/42MP, Thermo Fisher Scientific, Inc., Waltham, MA, USA). The vial cap was modified to assemble four sections of 1/16″ outer diameter Peek tubing (Trajan Scientific and Medical, Melbourne, Australia), which were used as outlets/inlets. The surrounding perimeter was sealed with self-leveling, heat-resistant silicone (Elastosil^®^ E43, Wacker Chemie AG, Munich, Germany) to ensure a watertight joint. The outlet was set at a distance from the vial bottom so that the culture volume was always maintained at 12 mL. The installation of custom check valves (Smart Products Inc., Mills River, NC, USA) at each inlet/outlet eliminated the possibility of upstream contamination and prevented possible backflow from downstream. In addition, they prevent leakage of fluids into the bioreactor as a consequence of possible insufficient clamping of the peristaltic pumps. Adding a polypropylene male Luer Lock to 1/16″ hose barb adapters (Masterflex^®^ MFLX4550100, Avantor Inc., Radnor, PA, USA) provided a quick and leak-proof connection. A piece of heat-resistant butyl rubber tape (W963 Plysafe, Plymouth Rubber Group, O Porriño, Spain) was employed to prevent the entrance of ambient light to the vial through the section not housed within the holder. The homogenization of the culture was carried out by 4.5 × 12 mm stir bars (Deltalab 19750, Deltalab Group, Rubí, Spain) driven by a 4-position magnetic stirrer with adjustable speed (MS-MP4, Witeg Labortechnik, Wertheim, Germany).

Liquid reservoirs were built employing borosilicate GL45 glass bottles of 2 and 5 L (Fisherbrand™ 15416123. Thermo Fisher Scientific, Inc., Waltham, MA, USA). Bottle caps were modified to connect 5 pieces of 1/16″ silicone tubing. To enable gas exchange with the atmosphere and to prevent pressure differences, both the culture vial caps and the reservoir caps were equipped with 0.22 µm filters (Millex^®^-GS, Merck, Tullagreen, Ireland).

The movement of fluids was enabled by the use of peristaltic pumps (Verderflex M045, Verder Liquids BV, Utrecht, The Netherlands). In total, four pumps were employed for each vial: one for the fresh media line, two for the fresh media with drug lines, and one more for the waste line.

The entire fluidic system, excluding the pumps, was housed in an incubator (Clear IC10-CE, OVAN, Badalona, Spain). The incubator was equipped with a heating and cooling system, which maintained a stable temperature within a 10–60 °C range. This feature represents an enhancement over other morbidostat designs, which are limited to operating at or above ambient temperatures.

### 2.2. Experimental Procedure on Morbidostat

To assess the efficacy of our morbidostat, we conducted an adaptive laboratory evolution (ALE) experiment employing sub-inhibitory concentrations of the antimicrobial DDAC as a source of selective pressure.

#### 2.2.1. Strain and Antimicrobials

*Escherichia coli* K12 (CECT 102) was obtained from Colección Española de Cultivos Tipo (CECT, Universidad de Valencia, Spain). Stock cultures were cryopreserved at −80 °C in 25% glycerol-supplemented meat-peptone broth (MPB) containing 0.5 g/L meat extract (Scharlau SL, Barcelona, Spain), 10 g/L (*w*/*v*) neopeptone (Bacto™. BD Biosciences, Franklin Lakes, NJ, USA), and 5 g/L NaCl (Emsure R, Merck KGaA, Darmstadt, Germany).

DDAC (95% purity, product code AB 172768) was purchased from ABCR GmbH & Co KG (Karlsruhe, Germany).

#### 2.2.2. Calibration of the Optical Subsystem

Before using the morbidostat, it is necessary to establish the correlation between the OD_600_ and the scattered light signal detected by the morbidostat phototransistors. In brief, an overnight *E. coli* K12 culture was prepared in MPB from a cryopreserved stock. Morbidostat vials were filled with 10.5 mL of fresh MPB. After 5 min of stabilization, the signal from each phototransistor was recorded. Then, 0.1 mL of cell culture and 1.4 mL of MPB were added to each vial. A 1.5 mL aliquot was then taken from each vial, and the OD_600_ was determined offline using a Thermo Multiskan Spectrum Spectrophotometer (Thermo Scientific, Waltham, MA, USA). This process was repeated until the OD_600_ reached a value of 0.4. Finally, the slope of the calibration curve between the light recorded by each phototransistor and the OD_600_ was set in the GUI.

During the calibration process, the culture conditions inside the vials were the same as those employed during the experiment. Thus, the temperature was kept at 37 °C to avoid temperature effects on the optical density measurement, and the agitation was maintained at 200 rpm to facilitate the homogenization of the culture.

#### 2.2.3. Sterilization of the Fluidic Subsystem

Before the start of the experiment, all the fluidic systems, including reservoirs, tubing, connectors, valves, filters, magnetic stirrers, and culture vials, were autoclaved (121 °C/20 min). The sterilization procedure differed from that previously reported in morbidostats, where sterilization was achieved by alternate rinses with ethanol, sodium hypochlorite, and sterilized water. After autoclaving, all parts were assembled in a laminar flow chamber, ensuring the sterility of the system.

#### 2.2.4. Morbidostat Experiment

Each culture vial was aseptically filled with 12 mL of MPB and inoculated with 200 µL of an overnight cell culture growing on the same medium. Vials were cleaned externally using cleaning wipes to ensure the absence of any particles that could affect the optical density reading. After that, each vial was placed in its respective holder in the same position in which it was calibrated, matching the small marks made on the holder and on the vial. This procedure minimizes the differences in the reading of the optical density value caused by possible irregularities in the glass.

To avoid delays in additions to the culture vial, the tubing was primed with the respective fluids (medium or medium-containing drug) by manually activating the corresponding peristaltic pumps through the GUI.

The following cultivation parameters were chosen: temperature: 37 °C, agitation: 200 rpm, OD threshold: 0.200, *V*: 12 mL, Δ*V*: 1 mL, Δ*t*: 12 min, *V*: 12 mL, and DDAC concentration in the Drug_A_ reservoir: 40 mg/L. The Drug_B_ reservoir was not employed. The morbidostat mode was initiated once the OD reached 0.100 to avoid excessive washout before the start of the exponential phase.

The adaptive evolution experiment on the morbidostat was terminated after three days. Thereafter, the sensitivity to DDAC of the evolved strains was evaluated.

#### 2.2.5. Evaluation of Sensitivity to DDAC of the Ancestral and Evolved Populations

The adaptive evolution experiment on the morbidostat was terminated after three days. Thereafter, the sensitivity to DDAC of the evolved strains was evaluated by determining the miminium inhibitory concentration (MIC) and the half maximal inhibitory concentration (IC_50_).

At the end of the ALE experiment, the entire volume of each morbidostat vial was transferred to conical tubes and centrifuged at 4000 rpm/15 min. The supernatant was discarded and replaced by 12 mL of 0.005 M KH_2_PO_4_ prior to a second centrifugation step (4000 rpm/15 min). The supernatants were discarded again, and pellets were resuspended in cation-adjusted Muller–Hinton broth (Sigma-Aldrich, St. Louis, MO, USA) to obtain an OD_600_ of 0.120, corresponding to 1 × 10^8^ CFUs/mL. From this, a subsequent 1:20 (*v*/*v*) cell suspension in Muller–Hinton broth was prepared. This cell suspension was employed as inoculum for the determination of MIC and in the growth kinetics assay. The cell suspension of the ancestral population was prepared in the same way from a 3-day culture in turbidostat mode. This was done to exclude any possible changes in DDAC sensitivity due to adaptation to continuous cultivation rather than stress due to the presence of DDAC. All of these operations and procedures were performed under sterile conditions.

The MIC values of the evolved populations from each vial were independently assessed by the broth microdilution method following the guidelines of the Clinical and Laboratory Standards Institute (CLSI) [13]. Briefly, a series of two-fold serial dilutions of DDAC were prepared in MHB in 96-well microtiter plates, resulting in concentrations ranging from 0.25 to 256 mg/L. The final cell concentration in each well was 5 × 10^5^ CFU/mL. The microtiter plates were incubated at 37 °C and 180 rpm for 24 h. Following incubation, the MIC values were determined to be the lowest concentration of DDAC, which completely inhibited visible bacterial growth. Each of the concentrations were tested in quadruplicate.

For the determination of IC_50_, a series of serial dilutions of DDAC was prepared in MHB using 96-well microtiter plates. In this case, the concentrations tested ranged from 2× MIC of the ancestral population (2× MIC_AP_) to a value below the 1× MIC_AP_, with a 10% decrement step variation: 4 mg/L (100% of the 2× MIC_AP_), 3.6 mg/L (90% of the 2× MIC_AP_), 3.20 mg/L (80% of the 2× MIC_AP_), 2.80 mg/L (70% of the 2× MIC_AP_), 2.40 mg/L (60% of the 2× MIC_AP_), 2 mg/L (50% of the of the 2× MIC_AP_, that is, the MIC_AP_), and 1.80 mg/L (90% of the MIC_AP_). Our experience has shown us that this approach is preferable to the conventional two-fold increase, which results in a very poor degree of resolution (growth vs. no growth) when working with quaternary ammonium compounds [14]. Each concentration was tested in triplicate for each of the evolved and ancestral populations. Plates were incubated at 37 °C and 180 rpm. Bacterial growth was spectrophotometrically determined at 600 nm at 0 and 24 h employing a microplate reader (Multiskan Spectrum, Thermo Scientific, Waltham, MA, USA). The 24 h OD_600_ values of each well were normalized as a percentage of inhibition respecting their respective controls (0 mg/L DDAC). Data were further fitted to the logistic model as follows [15] (Equation (5)):

(5) $$I=\frac{I_{m}}{1+\exp\left[r(IC_{50}-C)\right]},$$

where *I* represents the percentage of inhibition, $I_{m}$ is the maximum inhibition (value constrained to 100), *r* is the specific rate of the response (increment of the inhibition per unit of inhibition and per unit of DDAC concentration in L/mg), and *C* denotes the DDAC concentration (in mg/L).

Model parameters were estimated by minimizing the sum of squares of the residuals between the observed and predicted model values, using the non-linear least squares method (GRG non-linear) provided by the *Solver* add-in for Microsoft Excel^®^. The uncertainty in the estimation of the parameters was calculated employing Levie’s *SolverAid* macro [16].

## 3. Results and Discussion

The proposed design has been tested to demonstrate its robustness and reliability. Table 1 summarizes the differences between the design presented here and in previous works. The PLC gives the morbidostat capabilities that would be difficult to achieve using a desktop computer or hardware such as Arduino, which is more oriented to prototype development. OD values were determined every 5 s, resulting in a high temporal resolution compared to other morbidostat designs. Furthermore, incorporating two phototransistors and a noise filter enhances the reliability of the measurements while guaranteeing the correct functioning of the experiment in case of a malfunction of one of the two phototransistors.

### 3.1. ALE in Morbidostat

**Figure 6:**
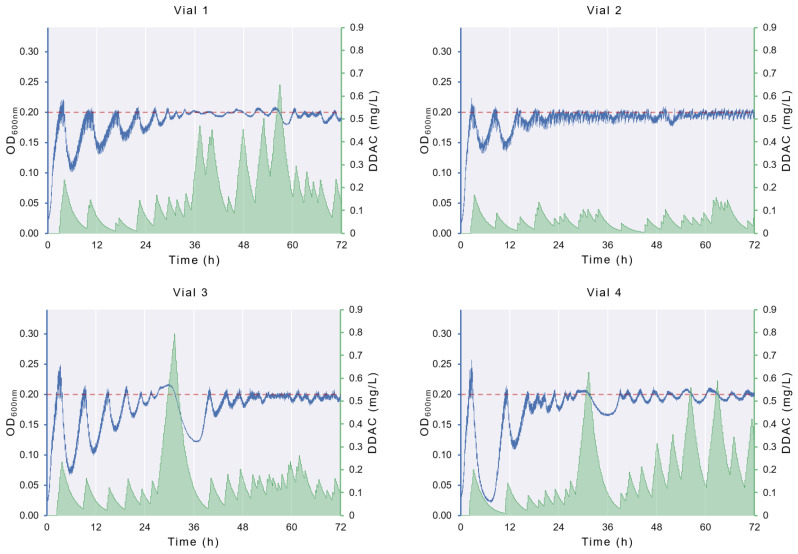
Growth profiles of *E. coli* populations on each of the morbidostat vials. Blue lines depict the OD_600_ values across the 72 h duration of the experiment, whereas green shaded area shows the variation in the DDAC concentration. The dashed red line symbolizes the OD threshold (0.2).

The growth profiles of *E. coli* in each of the morbidostat vials are shown in Figure 6. Variations in OD (blue line) after the addition of DDAC-containing culture medium reflect the effect of the antimicrobial on the population density. Over time, the decline in the population size following the addition of DDAC is less pronounced, evidencing the adaptative process. Nevertheless, variations exist between the vials. The evolved populations from vial 1 (EP1) and vial 2 (EP2) exhibited a more regular pattern, with a less pronounced initial decline and a subsequent plateau at 24–36 h, where the optical density (OD) rarely exceeded the threshold, and the DDAC has only a minor effect on the population size. The evolved populations from vial 3 (EP3) and vial 4 (EP4) exhibit another profile, with a pronounced decline following the initial DDAC addition and a second notable decrease at 36 h, coinciding with a peak in the DDAC concentration in the culture (green area). It should be stressed that, by definition of the morbidostat control law, DDAC is added only when the population exceeds the predefined threshold. Therefore, the differences in DDAC concentration in the different vials are also a consequence of differences in the population dynamics, with higher DDAC levels found in vials with better adapted populations.

The observed differences in the bacterial dynamics of the morbidostat can be attributed to fast and slow stochastic processes. The impact, due to differences in fast dynamics, is minor, usually reproducing similar behavior for short-term experiments. Common examples are randomness in population growth [19] and death due to antimicrobial factors [20]. Nevertheless, other stochastic dynamics drift the behavior of one vial with respect to others. Firstly, genetic drift and the randomness of mutations can result in the emergence of different subpopulations with a variable degree of sensitivity at different times [21]. Secondly, these discrepancies can also be attributed to the existence of a different subpopulation in the ancestral population with pre-existing mutations associated with DDAC susceptibility [22]. The founder effect, which occurs when the vials are inoculated, can alter the relative frequencies of each subpopulation or even eliminate them from the vial. Both scenarios were well documented in Leyn et al. [21], where the evolutive paths of six populations of *E. coli* exposed to triclosan in a morbidostat were determined by pre-existing mutations from the ancestral population, and de novo mutations emerged after 24 h of culture.

### 3.2. Changes in DDAC Susceptibility

Despite the differences in the dynamics of EP1 and EP2 compared to EP3 and EP4, all the evolved populations demonstrate a similar degree in DDAC susceptibility regarding the ancestral population.

The MIC of the evolved populations was 8 mg/L, which represents a four-fold increase in comparison to the MIC of the ancestral population (2 mg/L). The MIC value of the ancestral population was consistent with our previous findings [14,23], thereby validating the results. It is noteworthy that the MIC values of evolved populations were higher than the highest DDAC concentration to which they were exposed during the morbidostat experiment (Figure 6). This is consistent with the findings of Laument et al. [17], who observed that the exposure of *Neisseria gonorrhoeae* to sub-MIC concentrations of the disinfectant chlorhexidine digluconate in a morbidostat system resulted in a 10-fold increase in MIC.

**Figure 7:**
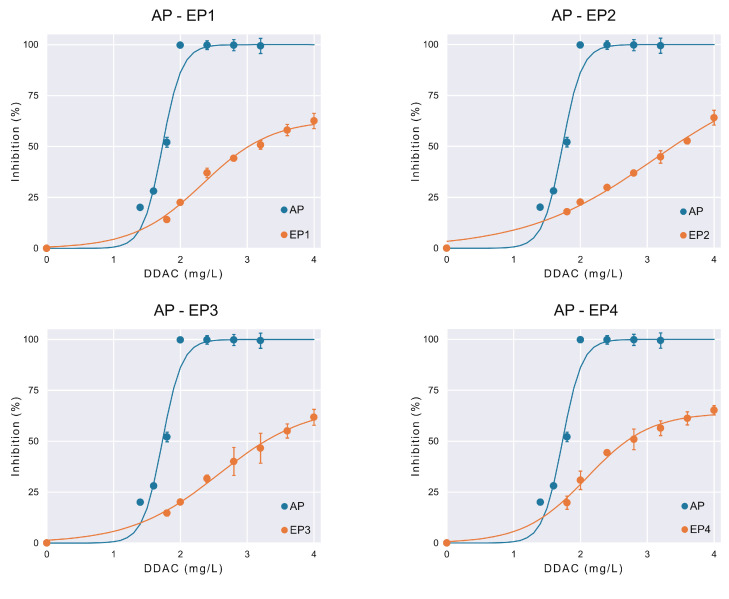
Comparison of the growth inhibition (%) achieved by different DDAC concentrations (mg/L) over ancestral (AP) and evolved (EP1, EP2, EP3, and EP4) *E. coli* populations. Inhibition percentages were normalized over the respective control (DDAC = 0 mg/L) for each population. Experimental data (depicted by markers) were fitted with the three-parameters logistic model (depicted by lines). Error bars show the standard deviation of the different four replicates.

The IC_50_ value was 1.73 ± 0.11 mg/L for the ancestral population, while evolved populations exhibited higher IC_50_ values, ranging from 2.09 ± 0.14 mg/L to 3.09 ± 0.94 mg/L. This increase in IC_50_ among evolved strains compared to the ancestral population highlights the significant adaptive changes and reflects lower DDAC susceptibility. The changes in IC_50_ are graphically depicted in Figure 7. Previously effective concentrations that were capable of completely inhibiting the growth of the ancestral population after 24 h were unable to even reduce the growth of the evolved populations by 50%. The logistic model fitted the inhibition data well, as indicated by high R-squared values across all populations (Table 2). Narrow confidence intervals, as seen in most parameters, indicate the high precision and reliability of the estimates, suggesting a good fit between the observed data and the model predictions. The analysis of other parameters also yields information of interest. The *r* parameter represents the rate at which inhibition increases with increasing DDAC concentrations. The ancestral population exhibited a *r* value of 6.83 ± 4.54 L/mg, indicating a steep inhibition curve. Evolved populations displayed lower *r* values (ranging from 1.03 ± 0.44 L/mg to 2.13 ± 0.14 L/mg), suggesting a more gradual increase in inhibition with higher DDAC concentrations, consistent with acquired resistance. The *I_m_* parameter represents the theoretical maximum inhibition achievable by DDAC. The ancestral population exhibited an *I_m_* of 100 ± 13.33%, indicating that DDAC concentrations higher than 2 mg/L could theoretically inhibit the entire microbial population. Evolved populations (EP1 to EP4) showed lower *I_m_* values (ranging from 63.26 ± 7.80% to 86.92 ± 37.37%), reflecting partial inhibition and the development of resistance mechanisms.

## 4. Conclusions

The PLC-controlled morbidostat developed in this study significantly improved the robustness, reliability, and real-time control capabilities of previous designs in the literature. This improvement addresses several limitations of previous designs, particularly in ensuring data validity and maintaining tight control over experimental conditions. To validate the system, we evolved *E. coli* under the stress of the common disinfectant DDAC. Note that adaptation to disinfectants is usually a much slower process than to antibiotics. Despite this difficulty, after three days, the minimum inhibitory concentration of the evolved population increased four-fold with respect to the ancestral population. The validation experiments, therefore, demonstrated the efficacy of the morbidostat in promoting the evolution of drug resistance and highlighted its potential for conducting adaptive laboratory evolution experiments.

The results in this work with *E. coli* and DDAC serve as a proof-of-concept. In future work, we will expand the range of microorganisms and antimicrobials tested. In addition, we intend to conduct direct comparative analyses with other morbidostat systems. This will offer crucial insights to enhance the design and further optimize adaptive laboratory evolution experiments.

## Figures and Tables

**Table 1:** Comparison of previous morbidostat designs with the proposed solution implemented into our device.

Aspect	Previous Works	Proposed Solution
Architecture	Monolithic, single process running in MATLAB or the like [2,3,7,17].	Hybrid, hierarchical design with well-defined functions distributed among the levels.
Long operation	Time-critical segments may suffer as the data sets grow throughout the experiment [5].	Real-time portions and subroutines are executed in the PLC, unaffected by data-acquisition growth.
Logic of the controller	Focus on Toprak’s morbidostat control law [2] with two conditions that cannot control the system when the antimicrobial effect has some latency.	Single-condition control law that ensures control even when the antimicrobial has a delayed effect.
Temperature control	Incubator chambers [2,3] or in-built heater solutions [18]. Cannot operate below ambient temperature.	Incubator equipped with a heating and cooling system. Stable temperature within a 10–60 °C range.
Sterilization of fluidic subsystem	Partially or totally chemical (successive washes with hypochlorite, ethanol, and distilled water) [2,3].	Totally physical (autoclaving). No risk of chemical residues.
Asepsis	Risk of external microbial contamination. Risk of contamination of the culture chambers and reservoirs by backflow [4].	Check valves reduce the risk of contamination in culture chambers and reservoirs.

**Table 2:** Numerical parameters from Equation (5), derived from the modeling of inhibition data on ancestral and evolved *E. coli* populations. Data are presented as mean ± confidence intervals (n = 4, α = 0.05). Goodness of fit (R^2^) is also shown.

Parameter	AP	EP1	EP2	EP3	EP4
*$I_{m}$* (%)	100 ± 13.33	63.26 ± 7.80	86.92 ± 37.37	67.49 ± 10.39	64.05 ± 5.85
*r* (L/mg)	6.83 ± 4.54	1.93 ± 0.71	1.03 ± 0.44	1.51 ± 0.45	2.13 ± 0.14
$IC_{50}$ (mg/L)	1.73 ± 0.11	2.33 ± 0.20	3.09 ± 0.94	2.57 ± 0.27	2.09 ± 0.14
$R^{2}$	0.971	0.992	0.993	0.995	0.993

## Data Availability

The original contributions presented in the study are included in the article. Further inquiries can be directed to the corresponding author.

## Abbreviations

The following abbreviations are used in this manuscript:

ALE	Adaptive Laboratory Evolution
COM	Communication
CFU	Colony-Forming Units
DAQ	Data Acquisition
DDAC	Didecyldimethylammonium Chloride
FBD	Function Block Diagram
FC	Flow Control
GUI	Graphical User Interface
IC_50_	Half-Maximal Inhibitory Concentration
MIC	Miminium Inhibitory Concentration
MPB	Meat-Peptone Broth
LED	Light-Emitting Diode
OD	Optical Density
PLC	Power Line Communications
PWM	Pulse Width Modulation
SCADA	Supervisory Control And Data Acquisition
STL	Standard Template Library
TCP	Transmission Control Protocol
VDC	Volts Direct Current
